# Does size matter? Trial management strategies to increase response rates. The assessment of physical questionnaire size on response rates (APS) sub-study

**DOI:** 10.1186/1745-6215-16-S2-P91

**Published:** 2015-11-16

**Authors:** Tracey Davidson, Gladys McPherson, Alison McDonald, John Norrie

**Affiliations:** 1 University of Aberdeen, Aberdeen, UK

## 

Trial management is dominated by the three Rs - recruitment, retention and responses. While much research goes into strategies to increase recruitment, retention and responses receive less attention. As trial managers we adopt several strategies to increase questionnaires response rates despite a lack of supportive evidence.

The majority of participant responses are collected via postal questionnaires sent to participants in a standard A4 size. Infrequently A5 size questionnaires are sent out, although this is usually for aesthetic reasons. Previous research found that length of questionnaire can influence response rates, but when the length of the questionnaire cannot be shortened is there anything else that can be done to maximise response rates? Limited previous research suggests that a patient's perception of questionnaire size may influence response rates. Given the importance of optimising these data, we aim to undertake an evaluation of sending either A4 or A5 sized questionnaires, with identical content, to participants.

We will discuss strategies used to increase response rates and the challenges, from a trial management perspective, of implementing these strategies both generally and in the APS sub-study. What to do about reminders? When do you stop the sub-study? How to decide who gets what and when?

We will also discuss the potential added benefits of this sub-study - cost saving in both printing and postage, and the knock-on “Green” effect of these measures - a metric that is recently been implemented at many institutions.

If size does matter, does one size fit all?

